# High-Throughput CRISPR Screening in Hematological Neoplasms

**DOI:** 10.3390/cancers14153612

**Published:** 2022-07-25

**Authors:** Raquel Ancos-Pintado, Irene Bragado-García, María Luz Morales, Roberto García-Vicente, Andrés Arroyo-Barea, Alba Rodríguez-García, Joaquín Martínez-López, María Linares, María Hernández-Sánchez

**Affiliations:** 1 Department of Translational Hematology, Instituto de Investigación Hospital 12 de Octubre (imas12), Hematological Malignancies Clinical Research Unit H12O-CNIO, CIBERONC, ES 28041 Madrid, Spain; rancos@ext.cnio.es (R.A.-P.); marimo13@ucm.es (M.L.M.); roberg09@ucm.es (R.G.-V.); albrod04@ucm.es (A.R.-G.); jmarti01@med.ucm.es (J.M.-L.); mlinares@ucm.es (M.L.); 2 Department of Biochemistry and Molecular Biology, Pharmacy School, Universidad Complutense de Madrid, ES 28040 Madrid, Spain; ibragado@ucm.es (I.B.-G.); andarroy@ucm.es (A.A.-B.); 3 Department of Medicine, Medicine School, Universidad Complutense de Madrid, ES 28040 Madrid, Spain

**Keywords:** CRISPR, hematological neoplasms, libraries, algorithms, resistances, vulnerabilities

## Abstract

**Simple Summary:**

High-throughput CRISPR screening provides an unbiased way for functional genomic studies in hematological malignancies. This approach has been used to study different blood cancers aiming to identify modulator genes for drug efficacy, therapeutic targets, synthetic lethal interactions, biomarkers of malignant transformation and the genetic determinants of immune evasion. In this review, we discuss the most relevant CRISPR screening studies in the field of hematology.

**Abstract:**

CRISPR is becoming an indispensable tool in biological research, revolutionizing diverse fields of medical research and biotechnology. In the last few years, several CRISPR-based genome-targeting tools have been translated for the study of hematological neoplasms. However, there is a lack of reviews focused on the wide uses of this technology in hematology. Therefore, in this review, we summarize the main CRISPR-based approaches of high throughput screenings applied to this field. Here we explain several libraries and algorithms for analysis of CRISPR screens used in hematology, accompanied by the most relevant databases. Moreover, we focus on (1) the identification of novel modulator genes of drug resistance and efficacy, which could anticipate relapses in patients and (2) new therapeutic targets and synthetic lethal interactions. We also discuss the approaches to uncover novel biomarkers of malignant transformations and immune evasion mechanisms. We explain the current literature in the most common lymphoid and myeloid neoplasms using this tool. Then, we conclude with future directions, highlighting the importance of further gene candidate validation and the integration and harmonization of the data from CRISPR screening approaches.

## 1. Introduction

The CRISPR–Cas (clustered regularly interspaced short palindromic repeats/CRISPR-associated) system, which was described as an adaptive immune system in prokaryotes, has become a powerful technology for genome editing [1]. This system relies on two main components: a guide RNA (sgRNA) and a CRISPR-associated (Cas) nuclease. The sgRNA is a specific RNA sequence that recognizes the target DNA region and directs the nuclease there for editing [2]. CRISPR-editing technology can easily introduce insertions or deletions to reproduce loss-of-function mutations, insert a specific sequence to generate point mutations, and with the use of alternative Cas enzymes can selectively activate or repress genes [3]. Therefore, this technology is an increasingly popular technique for genome engineering with significant improvements over the other gene editing technologies due to its capability to modify DNA sequences efficiently and accurately [4]. Furthermore, the CRISPR–Cas system has been scaled up to edit multiple genes in parallel, even up to the whole genome by the use of a library of sgRNAs [5]. Before the discovery of CRISPR, RNA interference (RNAi) libraries were regularly used for screening gene function as it is an efficient method that reduces gene expression at the mRNA level [6]. However, CRISPR technology is a more versatile approach because multiple screen formats can be carried out (knockout, interference, activation and epigenome screenings) [7]. In addition, this method has overcome the main drawbacks of RNAi-based screenings, showing stronger phenotypic effects, higher validation rates, and more consistent results with reproducible data and minimal off-targets effects [8,9]. Therefore, CRISPR screening has opened a broad range of applications in the era of precision medicine highlighting the discovery of drug targets and genes that contribute to drug resistance [10].

CRISPR screening typically involves several steps. First, the sgRNA library is created by synthetizing sgRNA oligonucleotides targeting the genes of interest and cloning them into a plasmid. Then, the plasmid pool is transduced into the target cells by viral transduction at a low multiplicity of infection to ensure that the vast majority of the infected cells carry only one plasmid copy. The transduced cells are usually selected by antibiotics. Next, the candidate genes are identified by next-generation sequencing (NGS)—using sequencing primers targeting sgRNAs—and comparing sequencing data from selected clones to an unselected control cell population; the sgRNAs that are enriched or depleted following selection will be analyzed. Finally, the candidate genes must be further validated by single in vitro or in vivo models [4].

The main purposes of CRISPR-based screens are identifying genes associated with drug resistance or essential genes that could be potential drug targets [11]. Depending on the aim of the study, either positive or negative screening can be used. In positive selection screens, a strong selective pressure is introduced, therefore, only cells with a relevant survival-enhancing perturbation will remain following selection. These screens are focused on resistant cells that proliferate, and very few hits are usually expected. Commonly, positive selection experiments are designed to identify perturbations that confer resistance to toxins, pathogens, or drugs. On the other hand, in negative screens, the goal is to identify perturbations that cause cell depletion during selection. Such perturbations typically affect genes that are essential for proliferation or survival, and therefore, become potential therapeutic vulnerabilities. Then, these essential genes can be found by comparing the relative frequency of each sgRNA between a late time point and an earlier one [12,13,14]. These screens can also detect synthetic lethal genes, as well as non-essential genes that become essential under a specific background. In this case, cells with a specific mutation and wild-type are infected with the sgRNA library, and synthetic lethal genes are identified based on sgRNA depletion in the mutant cells compared to the wild-type ones. Those provide alternative approaches for targeting a disease with a specific genetic alteration [15].

In the era of precision medicine, CRISPR–Cas screening has become a powerful tool to accelerate cancer research [4,16]. One of its main applications in oncology is to identify genotype-specific vulnerabilities with the aim of discovering novel potential drug targets [16,17]. Another application of CRISPR screening is investigating the underlying mechanisms of drug action, particularly in identifying genes that work synergistically with the drug or develop resistance to it [18]. Especially, this technology has been widely used to elucidate the mechanisms underlying drug resistance, since it is one of the main causes why patients relapse [19]. Furthermore, due to the known association between cancer and epigenetic alterations, epigenome-editing-based screening has emerged as a promising technique for underlying the regulatory networks that coordinate gene expression and their contribution to disease and drug response [20]. Several of these applications have been successfully translated to the hematology field, however, there is a lack of reviews focused on the wide uses of CRISPR screening in hematology. Therefore, in this review, we will highlight the main CRISPR-based approaches of high-throughput screenings that have been applied to study hematological malignancies. The recent developments achieved by this technology will be discussed with a special focus on the most common lymphoid and myeloid malignancies.

## 2. The CRISPR Screening Libraries Used for the Study of Hematological Malignancies

Before conducting a CRISPR screening experiment, researchers should keep in mind some considerations [21]. Concerning the libraries to choose, it is important to know which genetic modification we require for our research since there are different library types (knockout, activation, or inhibition). Beside this, the size of the library is also relevant as it has an impact on the number of genes that will be targeted (whole genome-wide libraries or custom libraries for a specific subset of genes, for example, a pathway). Another point to take into account is the species we are working with, because CRISPR libraries are designed specifically for the genome of a particular species and will only work in cells derived from that organism. Moreover, the format of the library is also relevant as CRISPR–Cas screen libraries can be conducted using arrayed or pooled libraries: arrayed screens are performed in multiwell plates with a single genetic perturbation per well; in pooled screens, sgRNAs are synthesized and then cloned to create a plasmid library that is after transduced in the target cells in the form of retrovirus or lentivirus [10]. Pooled libraries may be available in a one-plasmid system, in which Cas9 is included on the gRNA-containing plasmid, or a two-plasmid system in which Cas9 must be delivered separately. Interestingly, some of the pre-made sgRNA libraries are publicly available at Addgene (https://www.addgene.org/crispr/libraries/, accessed on 25 June 2022). In this section, we have described the most used CRISPR libraries for blood cancers and we have compiled the main information in Table 1.

### 2.1. Genome-Wide Libraries According to the Aim of the Study

#### 2.1.1. CRISPR Knockout

CRISPR knockout (CRISPRko) libraries produce a population of cells carrying mutations (insertions or deletions) that cause the silencing of specific protein-coding genes, rendering them nonfunctional. In general, knockout libraries can target up to 20,000 genes and usually contain from three to ten sgRNAs per gene, which prevents false negatives and ensures no missing gene hits. In the vast majority of these libraries, sgRNAs commonly target 5’ exonic regions, as frameshift mutations here increase the likelihood that a nonfunctional protein product is produced [22]. The most used system so far within this type has been the CRISPR Genome-scale Knockout (GeCKO) library, generated by Feng Zhang’s lab [5,23]. This library GeCKOv2 contains 123,411 sgRNAs split in two sub-libraries, in which each coding gene could be targeted by three sgRNAs [13], and is available as a one-plasmid system as well as a two-plasmid system [24]. It has been applied into the study of several hematological malignancies such as acute myeloid leukemia (AML), acute lymphoblastic leukemia (ALL), myeloma multiple (MM) and lymphoma (Table 1). Furthermore, they have developed another version for targeting mouse coding genes [23], which has been used for AML mouse cellular models [25]. There is another similar library available for the human genome, such as the one generated by Sabatini and Lander, with 10 sgRNAs per gene [26], from which different sub-pool sgRNA libraries have been generated and used in the study of hematological diseases [27]. Subsequently, other libraries with a lower number of sgRNAs have been generated seeking to increase the specificity of the sgRNAs, in order to avoid false negative results [28]. These include the Toronto Knockout (TKO) library, generated by Moffat’s lab [17,29,30] and the Brunello library generated by Doench and Root [28]. Both libraries contain around 70,000 sgRNAs and four sgRNAs per gene [28]. Recently, the comparison of different CRISPR libraries has determined that Brunello showed the highest depletion of sgRNAs targeting essential genes, even with a lower number of sgRNAs per gene [31]. This library has also been broadly applied in the study of blood tumoral disorders such as chronic lymphocytic leukemia (CLL), AML, MM, and lymphoma (Table 1). Alternatively, the Gattinara library also developed by the Doench lab, has been used for assays with a limited number of cells since it contains around 40,000 sgRNAs with two sgRNAs per gene.

Doench and Root have collaborated as well in the development of the Avana library [28]. On the other hand, the Human Improved Genome-wide Knockout CRISPR developed by Tzelepis [32], targeting 18,010 genes, has been used to study AML and lymphoma (Table 1). Unlike the above-mentioned libraries which are Cas9-dependent, the BARBEKO library has recently developed by Wei’s lab to perform knockouts through the use of CRISPR cytosine base editors (CBE) perturbing gene start codons, splice sites, or introducing premature termination codons, without the use of Cas9 [33]. This approach has been tested in K-562, which is a cell line of chronic myeloid leukemia (CML).

#### 2.1.2. CRISPR Activation

CRISPR activation (CRISPRa) is an optimized method for specific gene overexpression using an inactivated Cas9 (deadCas9 -dCas9-) with added transcriptional activators to upregulate target genes within their native context, overcoming other technologies such as those based in the used of cDNA and ORFs [34]. dCas9 with activators are led by sgRNAs towards the promoter or transcriptional start sites of specific genes, allowing for gene modulation and overexpression. CRISPRa libraries are based on this editing approach [10].

Although all the libraries use a similar mechanism targeting dCas9 activators towards specific gene fragments, the components involved in each mechanism may vary from one library to another. The CRISPRa library, published by the Weissman lab (Gilbert et al.), has used the SunTag-VP64 system which contains the activator scFV-VP64 [35]. This library has been used in the CML cell line K-562 [36,37] and to study lymphoma [38]. The library, developed by Feng Zhang’s lab, has been based on the Synergistic Activation Mediator (SAM) containing the activator dCas9–VP64 and an activator complex (MS2–p65–HSF1) allowing for robust transcriptional activation [34]. In addition, Root and Doench have developed the Calabrese library which contains the p65–HSF1 transcriptional activation domain [31]. Whereas the Weissman and Calabrese library contains more than 100,000 sgRNAs with 5–10 sgRNAs per gene and 3–6 sgRNAs per gene, respectively [31,37], the SAM library only contains around 70,000 sgRNAs using three sgRNAs per gene [34]. One study comparing the Calabrese and SAM library has reported that Calabrese can be more effective, revealing a higher number of gene hits involved in drug resistance [31]. Whereas no studies have been published so far using Calabrese library to study hematological diseases, the SAM library has been used to test the efficacy of drugs in lymphoma [39] and AML [40].

**Table 1:** Main information of the CRISPR human libraries used in hematological disorders.

Library-Name	Library Type	Library Size	Total sgRNAs (Targeted Genes)	gRNAs Per Gene	Addgene Reference	Used in Hematology	Aim of the Study
Sanjana et al. [23]—**GeCKO**	Knockout	Genome-wide	123,411 (19,050 genes and 1864 miRNAs)	6	#1000000048	**ALL** [41,42,43], **AML** [44,45,46,47,48,49], **CML** [50], **HL** [51], **NHL** [52,53,54], **MDS** [55], **MM** [56,57,58,59,60]	Drug resistance and sensitivity, therapeutic vulnerability, synthetic lethality
Doench et al. [28]—**Brunello**	Knockout	Genome-wide	76,441 (19,114)	4	#73179	**AML** [61,62], **CLL** [63], **CML** [64], **NHL** [65,66,67,68], **MM** [69]	Drug resistance and sensitivity, therapeutic vulnerability, synthetic lethality
Tzelepis et al. [32]—**Human improved genome-wide library**	Knockout	Genome-wide	90,709 (18,010)	~5	#67989	**AML** [32,70,71,72,73,74,75], **NHL** [76]	Drug resistance and sensitivity, therapeutic vulnerabilities, synthetic lethality
Doench et al. [28]—**Avana**	Knockout	Genome-wide	73,782 (18,547)	4	NA	**ALL** [77], **AML** [78]	Drug sensitivity, therapeutic vulnerability
Hart et al. [17]—**Toronto KnockOut (TKO)**	Knockout	Genome-wide	176,500 (17,661)	6	#1000000069	**CLL** [79]	Synthetic lethality
Jaiswal et al. [80]	Knockout	Custom	268 (36 RBP genes)	~4	NA	**ALL** [80]	Therapeutic vulnerability
Gabra M et al. [81]—**miRKO library**	Knockout	Custom	6835 (1795 miRNAs)	3 to 4	NA	**AML** [81]	Therapeutic vulnerability
Lin S. et al. [82]	Knockout	Custom	1320 (~200)	6	NA	**AML** [82]	Therapeutic vulnerability
Liss et al. [83]	Knockout	Custom	NA	NA	NA	**AML** [83]	Therapeutic vulnerability
Lin C.H. et al. [84]	Knockout	Custom	NA	NA	NA	**AML** [84]	Therapeutic vulnerability
Lin K.H. et al. [85]	Knockout	Custom	11,610 (2322)	5	NA	**AML** [85]	Therapeutic vulnerability
Ott et al. [86]	Knockout	Custom	~3500 (147 TFs)	~7	NA	**CLL** [86]	Therapeutic vulnerability
Kazimierska et al. [64]—**MYC-CRISPR library**	Knockout	Custom	46,354 (24,981)	~2	#173195	**CML** [64]	Therapeutic vulnerability
Han et al. [87]–**Double-sgRNA library**	Knockout	Custom	~490,000 double-sgRNAs (21,321)	up to 9	NA	**CML** [87]	Synthetic lethality
Wei et al. [88]—**Ubiquitin regulator-focused library**	Knockout	Custom	~1300 (800)	10	NA	**HL** [88,89]	Drug sensitivity, therapeutic vulnerability
Mo et al. [90]	Knockout	Custom	19,011	4 to 8	NA	**NHL** [90]	Drug resistance
Bohl et al. [91]	Knockout	Custom	745 (177)	~4	NA	**MM** [91]	Drug resistance, drug sensitivity
Shen et al. [92]	Knockout	Custom	30 (3)	10	NA	**MM** [92]	Therapeutic vulnerability
Wang et al. [26]—**Kinase gRNA library**	Knockout	Custom	73,151 (7114)	10	#51044	**ALL** [27]	Drug sensitivity
Gilbert et al. [35]—**CRISPRi**	Interference	Genome-wide	206,421 (15,977)	10	#62217	**NHL** [38]	Drug resistance
Gilbert et al. [35]—**CRISPRa**	Activation	Genome-wide	198,810 (15,977)	10	#60956	**AML** [40], **NHL** [38]	Drug resistance
Konermann et al. [34]—**SAM**	Activation	Genome-wide	70,290 (23,430)	3	#1000000078	**AML** [39]	Drug resistance
Bester et al. [40]— **CaLR**	Activation	Custom	88,444 (14,701 lncRNA genes)	~4	NA	**AML** [40]	Drug resistance

NA: Not available.

#### 2.1.3. CRISPR Interference

CRISPR technology can be also used to inactivate gene expression using dCas9 fused with repressor domains [93]. CRISPR inactivation (CRISPRi) libraries can simultaneously knockdown the expression of large sets of genes [10]. Among them, the Weissman laboratory has developed a CRISPRi library based on the dCas9–KRAB mechanism, which consists of a fusion between dCas9 and the transcriptional repressor KRAB (Kruppel-associated box domain) [37]. In addition, Root and Doench have also developed the Dolcetto library, based on the KRAB–dCas9 mechanism. Whereas the Weissman library contains 5–10 sgRNAs per gene, the Dolcetto library only contains three to six sgRNAs each gene [31]. Interestingly, the efficiency of CRISPRi library has been compared with CRISPRko libraries observing a very similar number of hits [31]. Within the group of interference libraries, only CRISPRi (developed by Weissman laboratory) has been applied to the study of hematological diseases [36,38].

### 2.2. Custom CRISPR Libraries

CRISPR libraries can be reduced by restricting the number of genes and limiting them to those genes that are relevant to our study (genes differentially expressed in another experiment, genes with a particular function or involved in a specific pathway…). Then, custom CRISPR screens, which contain fewer gRNAs and are less expensive, do not require such a huge number of cells and high sequencing depth as genome-wide libraries. It is worth mentioning that there are several methods to design this type of libraries such as CORALINA [94], CRISPR library designer CLD [95] and Green Listed [96], which has a web tool to rapidly extract a subset of sgRNAs from other public genome-wide libraries (http://greenlisted.cmm.ki.se/, accessed on 25 June 2022).

In the field of hematology, several custom libraries have been used to study specific molecular pathways or a particular set of genes that play a relevant role in the development of the disease or during treatment. Of note, a custom library containing sgRNAs targeting 36 highly regulated RNA-binding protein (RBP) genes in B-ALL was designed to identify which ones are essential in B-ALL [80]. Other libraries targeting only kinases or genes involved in DNA damage response have detected gene modulators whose disruption could enhance the sensitivity of drugs such as asparaginase or PARP inhibitors (PARPi), respectively [27,97].

In other studies, custom libraries targeting non-coding RNAs such as microRNAs (miRNAs), long non-coding RNAs (lncRNAs) and circular RNAs (circRNAs) have been developed to determine the role of these key post-transcriptional modulators of gene expression in cancer [98,99,100]. Interestingly, the use of a miRNA-only knockout (miRKo) library has allowed the identification of essential miRNAs in AML [81]. In addition, the combination of two custom libraries targeting lncRNAs to perform CRISPR knockout and CRISPRi have evaluated the impact of lncRNAs in the cellular growth of CML K-562 cells [60]. By contrast, another library to perform CRISPRa of lncRNA (CaLR) has helped to identify, for the first time, which lncRNAs are involved in the response to cytarabine treatment in AML [40].

An alternative approach of CRISPR technology is the editing of the epigenetic landscape to control gene expression without cleaving the DNA sequence [101]. In this case, dCas9 can be fused to different epigenetic modifiers to edit the methylation state of cytosines in a gene’s promoter or to induce histone acetylation or demethylation; the sgRNAs target a specific promoter or enhancer for the gene of interest [101]. In order to identify the enhancer and regulatory elements of proximal and distal genes, the CRISPR–Cas9-based epigenomic regulatory element screening (CERES) has been developed to carry out parallel loss- and gain-of-function experiments to study the function of regulatory regions in their native genomic context using the dCas9–KRAB mechanism, dCas9–p300 as an activator, and sgRNA-targeting DNase I hypersensitive sites surrounding a gene of interest [20]. Furthermore, the CRISPR-based programmable epigenome editor protein CRISPRoff, (dCas9 fused with KRAB machinery and the protein domains D3A and D3L) and CRISPRon (dCas9 in combination of VP64, p65-AD and Rta transactivator domains) have shown mechanisms of heritability after cell division [102].

### 2.3. In Vivo CRISPR Screenings

Although the vast majority of CRISPR screening studies published to date use in vitro cell cultures, in recent years, in vivo CRISPR screens have been carried out in order to recapitulate the complexity of a living organism [103]. The main advantage of in vivo CRISPR screenings is the possibility to evaluate the edited cells within their natural niche in the presence of the microenvironment [15]. Direct in vivo screens are able to study the effect of genetic alterations within the corresponding tissue environment since the perturbation reagents are delivered directly to the cells of interest in a living animal. The main purpose of this mutagenesis is to uncover cancer driver genes and unique cancer dependencies in the context of the tumor microenvironment [104,105,106].

On the other hand, indirect in vivo screens consist on the transplantation of pre-edited cells by the sgRNA library into an animal [107]. In solid tumors, this application has been mainly used to identify gene hits that can functionally drive the tumor growth and the metastasis process [108]. In the hematology field, this strategy has been applied to monitor the clonal evolution in MM through the designing of a targeted library targeting *HMGA1*, *PA2G4* and *TRIM28*, which are involved in MM progression [92]. Indirect in vivo CRISPR screening has also been used to study tumor progression in myeloid leukemias, helping to identify the double-stranded RBP Stau2 as a critical dependency of this malignance [109], and to identify AML-enriched dependencies [82]. To evaluate lymphomagenesis, a CRISPR loss-of-function screen targeting murine orthologs of genes infrequently mutated in Burkitt lymphoma (BL) was used [110]. However, it is worth taking into account that in vivo genome editing presents some limitations, which could explain the lack of studies using this approach. Among them, delivery of the CRISPR machinery could be inefficient in some tissues of living animals; the number of edited cells could be lower which could reduce the number of relevant hits comparing to in vitro screens; the immune system could impact clonal dynamics adding noise to the screening, requiring multiple replicates [21]. These limitations require further research in order to improve the applications of in vivo CRISPR screenings.

## 3. Bioinformatic Tools in CRISPR Screening of Hematological Disorders

### 3.1. Algorithms

The variety of screening platforms have caused the development of different algorithms for CRISPR analysis [111]. In a typical screen workflow, after the viral delivery of the library and the selection for transduced cells, deep sequencing of PCR-amplified genomic DNA from selected clones and unselected ones is performed to quantify the representation of sgRNAs and evaluate as to whether they are enriched or depleted. Then, sequence reads must be mapped against the original sgRNA library, which stores the gene annotation to each sgRNA allowing gene quantification. Finally, statistical analysis is needed to identify significant gene hits likely to be relevant to the phenotype of interest [112].

Most algorithms aim to quantify the sgRNA effects individually and then to aggregate effects from sgRNAs that target the same gene to infer gene effect. The input data usually contain a matrix populated with the raw read counts of sgRNAs, where the columns are samples/replicates in the CRISPR screen and rows are individual sgRNAs and the gene identities which map sgRNAs. The comparison of sgRNA abundance between conditions (e.g., beginning/end of an experiment or treated/untreated cells) is the key step to identify hit genes. For this purpose, each algorithm uses different methods [111].

In the CRISPR screening of blood cancers, a great variety of bioinformatic tools have been carried out to analyze sequencing data (Table 2). The Model-based Analysis of Genome-wide CRISPR–Cas9-Knockout (MAGeCK) algorithm has been the most used and cited algorithm (Table 2) because it is well-documented, versatile, exhaustive and constantly updated. Briefly, MAGeCK uses a median-based normalization approach with a negative binomial distribution (similar to DESeq2 [113]) and a robust rank aggregation (RRA) method to prioritize sgRNA, genes or pathways between different experimental conditions. Its output is a list of genes with their False Discovery Rate (FDR) [114]. It has been demonstrated that obtaining high-quality data in CRISPR high-throughput studies allows one to obtain concordant results independently to the selected algorithm [115]. In this context, MAGeCK–VISPR is an updated version that has mainly added quality control parameters at different levels and visualization tools [116]. Another recently developed tool, MAGeCK–Flute, integrates MAGeCK and MAGeCK–VISPR providing new quality control and downstream analysis functions [117].

Other algorithms such as the Bayesian Analysis of Gene EssentiaLity algorithm (BAGEL) [126] and STARS [28], which are based in supervised learning and gene ranking methods, respectively, have been also used in CRISPR screenings of hematological neoplasms (Table 2). BAGEL has been only applied in leukemia screenings [128,129] whereas STARS has been used with leukemia [125], myeloma [56] and lymphoma [63] models.

Taking into account that copy number (CN) events are usual in hematological malignant diseases [135] and that recent studies have shown the increase of false positive relevant genes in CRISPR screening due to CN, some algorithms have been designed to solve this problem such as CRISPy [136], CRISPRcleanR [137], and CERES, which has been implemented in the Broad DepMap project [131].

Whereas many CRISPR screens have been performed to analyze only a single ‘end point’, it can be informative to evaluate multiple time points to obtain dynamic changes over the course of the screen. Multiple screens can be also carried out using multiple cell lines or multiple conditions (e.g., several treatments). In this context, MAGeCK–VISPR [116], the joint analysis of CRISPR–Cas9 knockout screens algorithm (JACKS) [132], and CERES [131] are the best options because they are designed to deal with multiple screening experiments.

Single-cell-based CRISPR screening [138,139,140] has emerged as a powerful strategy in cancer [141,142,143]. These techniques combine CRISPR screening and scRNA-seq to obtain a comprehensive readout of the perturbations introduced at cellular level [144]. Due to its novelty, few computational tools are available, one being single cell MAGeCK (scMAGeCK) [141], which is a version of MAGeCK for single-cell studies (Table 2), and Gene expression clustering [145], two of the well-known algorithms [146]. In addition, other methods such as Seurat [147] have been used in hematology to analyze single-cell data coming from CRISPR screens [148]. The application of this technology in hematological research provides new opportunities to gain relevant insight into this field.

### 3.2. CRISPR Screening Databases

CRISPR screening experiments have accumulated a huge amount of data aiming to relate genotype and phenotype information. To facilitate access to this information, several public databases have been developed in the last few years. The Cancer Dependency Map (DepMap) is a collaborative project developed by the Broad Institute and the Sanger Institute aiming to identify cancer vulnerabilities through the in vitro study of genetic dependencies in cancer cell lines [149,150].

The DepMap portal (https://depmap.org/portal/, accessed on 25 June 2022) integrates three large-scale projects of CRISPR–Cas9 loss-of-function screens (the Broad Achilles screens [151], Sanger CRISPR screens [152] and GeCKO libraries [131]). This repository allows us to discover genetic and pharmacological dependencies in human cancer cell lines, offering omics data at different levels (gene expression, CN events, mutations, methylation and protein data) from cell lines as well as from compound viability screens. This database is updated every 6 months and has nearly 2000 cancer cell lines with a total of 169 coming from hematopoietic and lymphoid tissues, 129 from lymph nodes and 63 from bone marrow. Specifically, in these groups, 137 cell lines belong to leukemia, 110 to lymphoma and 35 to myeloma. Moreover, the DepMap portal provides us valuable information of cell lines associated with diseases of interest and lists of genes or compounds specifically enriched or used in hematological diseases. In addition, a tool to compare gene expression/mutational patterns between two predefined cell line groups and to correlate pairs of genes/compounds in specific contexts is freely available and could be useful to extract relevant genetic information for hematologic research.

The Project Score from Sanger DepMap (https://score.depmap.sanger.ac.uk/, accessed on 25 June 2022) is focused on developing genetic screens to identify cancer dependencies in order to prioritize new target gene candidates for cancer therapy [153]. Its web-portal data from genes, cancer cell models, or tissue types have been generated with their own methodology [151]. Among the total of 914 cancer cell lines, the Sanger DepMap has 86 hematopoietic and lymphoid tissue cell lines. Specifically, 38, 23, 21 and 4 cell lines belong to leukemia, lymphoma, myeloma and other blood cancers, respectively. Furthermore, this portal contains a list of essential genes grouped by disease/cell line types with the possibility of excluding pan-cancer important genes. Additionally, they provide a list of scored targets which could be helpful to nominate candidate targets in hematology and their tractability for drug development.

Strategies for cell infection, library design, screen duration or sequencing methods deviate between the two aforementioned projects. Whereas the Sanger project uses CRISPRcleanR [137] and BAGEL2 [127] to analyze data obtaining a “fitness score” for genes in each cell line together with biomarker information (CN variations and point mutations) to prioritize targets, the Broad Sanger Project uses algorithms such as Chronos [154] and CERES [131], which generate a different metric called the “gene effect”. In addition, a subset of cell lines are shared by the two projects while both of them also have their own cell lines. Therefore, the high amount of data found in these datasets needs to be curated, harmonized and unified to facilitate their use and understanding by the scientific community. In this sense, Sanger Depmap and Broad Depmap projects are trying to unify the information in order to establish a more comprehensive cancer dependency map [150].

Other used but less-relevant databases and repositories in CRISPR screening with data related to hematological disorders are GenomeCRISPR [155] (http://genomecrispr.dkfz.de/#!/, accessed on 25 June 2022), PICKLES [156] (https://pickles.hart-lab.org/, accessed on 25 June 2022), BioGRID ORCS [157] (https://orcs.thebiogrid.org/, accessed on 25 June 2022) and iCDBS [158] (https://www.kobic.re.kr/icsdb/, accessed on 25 June 2022). 

## 4. Mechanisms of Drug Resistance Uncovered by CRISPR High-Throughput Screening in Hematological Malignancies

**Figure 1:**
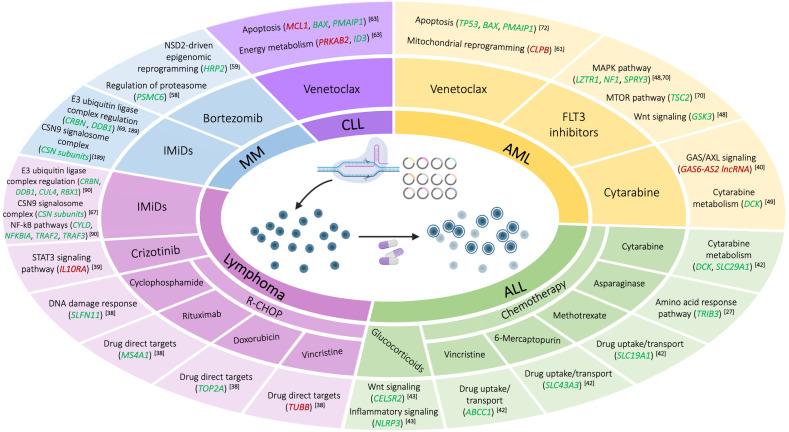
Drug resistance mechanisms identified by CRISPR screening in the main hematological malignancies, reviewed in this work. Diseases are in the inner circle (AML, acute myeloid leukemia; CLL, chronic lymphoblastic leukemia; MM, multiple myeloma; ALL, acute lymphoblastic leukemia); therapies are in the middle circle (IMIDS, immunomodulatory drugs; R-CHOP, rituximab, cyclophosphamide, doxorubicin, vincristine, and prednisone); main pathways, in which each set of validated genes is involved, are in the outer ring. In green, the loss of the gene produces the gain of resistance; in red, the overexpression of the gene produces drug resistance.

Despite the continuous advances in targeted therapies into hematological malignancies, drug resistance and their failure to obtain durable response remains one of the main challenges for clinical management. The development of drug resistance limits the drug effectiveness with a great impact on disease progression and outcomes in patients. It is difficult to predict the mutations associated with poor response. In this context, CRISPR genome-wide screening tools have proven to be quite useful in uncovering treatment failures. These approaches have provided a fast and effective way to search for genetic alterations related to drug resistance in hematological malignancies. In this section, we discuss the recent works focused on the most common lymphoid and myeloid neoplasms using this tool (Figure 1).

### 4.1. Lymphoid Neoplasms

#### 4.1.1. Lymphoma

Lymphomas are a heterogenous group of hematological malignancies traditionally divided into Hodgkin’s lymphoma (HL) and non-Hodgkin’s lymphoma (NHL), which accounts for about 90% of all lymphomas [159,160]. Diffuse large B-cell lymphoma (DLBCL) is the most common NHL and almost half of the patients experience resistance to the standard care treatment R-CHOP (rituximab, cyclophosphamide, doxorubicin, vincristine and prednisone) [161,162]. Interestingly, genome-wide CRISPRi and CRISPRa libraries have revealed the mechanisms of single drug resistance, obtaining consistent gene hits associated with known mechanisms of drug action. Specifically, knockdown of *MS4A1* (encoding CD20) has conferred resistance to rituximab and *TOP2A* (encoding topoisomerase II) to doxorubicin, whereas the overexpression of *TUBB* (encoding b-tubulin) has conferred resistance to vincristine. Meanwhile, cyclophosphamide resistance has been related to multiple genes involved in the DNA damage response, such as *SLFN11*. Of note, the constituents of R-CHOP have non-overlapping resistance mechanisms, and therefore, this low-cross-resistance could be a key attribute of the curative R-CHOP regimen in DLBCL patients [38].

To overcome the limitations of the standard chemotherapy, new drugs have been developed, such as immunomodulators and cereblon-modulating agents [163] as well as novel inhibitors [164]. Regarding immunomodulators and cereblon modulator agents, genome-scale CRISPR–Cas9 screenings, performed in cell lines of DLBCL and primary effusion lymphoma (PEL), have shown that not only is the loss of well-defined members or regulators of the E3 ubiquitin ligase complex (*CUL4*, *DDB1*, *RBX1*, *CRBN*) which interacts with cereblon, possibly associated with resistance, but also that of other genes involved in canonical and noncanonical NF-κB pathways (*CYLD*, *NFKBIA*, *TRAF2*, or *TRAF3*) or COP9 signalosome (CSN) subunits [67,90].

Crizotinib, which is a novel small inhibitor of anaplastic lymphoma kinase (ALK) fusion protein, has been used in ALK-positive lymphomas, reducing toxicity and side effects [165]. However, a subset of patients has progressed within the first months of treatment [166]. The combination of genome-wide CRISPR activation and knockout screens in ALK-positive cell lines have shown that crizotinib resistance could be mainly driven by an aberrant upregulation of interleukin 10 receptor subunit alpha (*IL10RA*). Taking into account that IL10RA expression has not been correlated with standard chemotherapy response, the combination of crizotinib plus chemotherapy could overcome ALK inhibitor resistance [39]. Another emerging anti-lymphoma therapeutic strategy has been the inhibition of the eIF4A RNA helicase with silvestrol and its related compounds [167]. CRISPR screens have identified three negative NRF2 regulators (*KEAP1*, *CUL3*, *CAND1*) whose inactivation might be sufficient to cause drug resistance in murine lymphoma cell lines [168].

#### 4.1.2. Acute Lymphoblastic Leukemia

ALL is a hematologic malignancy characterized by the cancerous transformation of immature lymphoid progenitor cells. Effective treatments have been based on intensive chemotherapy, especially for younger patients. However, those who have relapsed have shown worse clinical outcomes as a result of chemotherapy resistance [169]. CRISPR–Cas9 knockout screenings following the exposure to seven chemotherapy drugs have not only confirmed genetic drivers of chemoresistance previously observed in ALL patients (disruption of *TP53* [170], *NT5C2* [171], *PRPS1* [172] and *CREBBP* [173]) but have also uncovered other novel alterations not reported yet [42]. Interestingly, genes implicated in drug transport have been commonly involved in chemotherapy resistance such as *ABCC1* (vincristine transporter), *SLC19A1* (mediator of cellular methotrexate uptake), *SLC43A3* (membrane transporter of 6-Mercaptopurine) or *SLC29A1* (mediator of cytarabine cellular import). Additionally, genes implicated in other pathways such as the drug metabolism (*DCK* for cytarabine or *ASNS* for L-asparaginase) or drug targets (*TOP2B* or *TOP2A* for daunorubicin) have been determined. It is worth mentioning that some top-scoring gene hits have been commonly involved in multiple drug resistance (*CAD* for methotrexate and L-asparaginase, *EIF31* for cytarabine and 6-mercaptopurine, *HSPE1* for cytarabine and daunorubicin, *NAA10* for vincristine and L-asparaginase, among others) [42]. Another CRISPR screening approach using a different ALL cell line has highlighted that the loss of the pseudokinase Tribbles 3 (*TRIB3*), a proapoptotic target gene, could also contribute to asparaginase resistance [27]. Since chemotherapy is usually combined with glucocorticoids to enhance its efficacy [174], the identification of the genomic and epigenomic determinants of glucocorticoids resistance can gain insight towards the improvement of ALL treatments. Genome-wide CRISPR screening has confirmed some genes previously related to glucocorticoid resistance in ALL patients such as *NLRP3* [175] and *SMARCA4* [176] and it has also allowed researchers to reveal 14 genes not previously associated [43]. Among them, *CELSR2* was the top candidate in which low expression levels have been related with an upregulation of anti-apoptotic *BCL2*. Then, glucocorticoid resistance caused by *CELSR2* knockdown could be mitigated by BCL2 inhibitors such as venetoclax [43]. The tyrosine kinase dasatinib has been recently approved to be used in combination with chemotherapy for a subgroup of ALL patients with *BCR-ABL1* fusion [177].

More recently, the HDAC inhibitor panobinostat, which has recently approved for multiple myeloma, has shown anti-leukemia effects in ALL [178,179]. As a further step, the genome-wide CRISPR technique has elucidated mitochondrial activity as the driver of panobinostat resistance [41]. Particularly, *SIRT1* expression activates mitochondrial activity and sensitizes ALL patients to panobinostat, showing *SIRT1* to be a potential biomarker [41].

#### 4.1.3. Chronic Lymphocytic Leukemia

CLL is a well-defined lymphoid neoplasm and one of the most prevalent types of leukemia around the world [180]. During the last decade, there has been an impressive explosion of new approaches based on targeted therapies such as BCR and BCL2 inhibitors for CLL patients [181,182]. Despite their potent clinical activity in even high-risk CLL patients, disease progression on these novel drugs represents still an emerging therapeutic challenge [183]. Loss-of-function CRISPR screening together with an open reading frames library have been used to identify the genes involved in resistance to venetoclax (BCL2 inhibitor). The top candidate drivers of venetoclax resistance were BCL2 family members (*BCL2L11*, *MCL1*, *BAX*, *BAK1* and *PMAIP1*), lymphoid transcription factors (ID3) and genes involved in PKA/AMPK signaling (*PRKAR2B*, *PRKAA2*) [63]. The venetoclax resistance involves both the reprogramming of the biology of the outer membrane of the mitochondria, leads to expression changes in BCL-2 family members, affecting mitochondrial function, and increases OXPHOS activity in inner membrane of the mitochondria. Similar findings were also achieved by the functional characterization of venetoclax-resistant cell lines and molecular characterization of venetoclax-relapse CLL patients [63].

#### 4.1.4. Multiple Myeloma

MM is a neoplastic proliferation of plasma cells [184]. During the last decades, the outcome of MM has significantly improved with the use of proteasome inhibitors (PI), specifically bortezomib (BTZ) [185]. Despite the fact that almost all patients can achieve good responses, most of them eventually develop drug resistance over time [185]. Using CRISPR screening, the proteasome regulatory subunit *PSMC6* has been described as the most prominent gene associated with BTZ resistant [58]. Interestingly, this gene and other members of the PSMC family, which are located in in the base region of the proteasome 19S regulatory particle, have been also related with BTZ resistance in other CRISPR and shRNA screenings [57,186]. Of note, mutations and overexpression of *PSMB5* (a catalytic subunit located at the proteasome 20S core) have been correlated with BTZ resistance [187]. Alternatively, a CRISPR ex vivo screening has demonstrated the loss of *HRP2* as another gene determinant of BTZ resistance. Interestingly, the disruption of *HRP2* could lead to an epigenomic reprogramming in BTZ resistant cells [59].

Another common therapy in MM is the use of immunomodulatory imide drugs (IMiD), such as lenalidomide and pomalidomide, which have been significantly improved the survival of MM patients [188]. The genome-wide CRISPR–Cas9 knockout screenings have identified the *CRBN* (adaptor for CRL4^CRBN^ E3 ligase complex) as the top-ranking mediator of both lenalidomide or pomalidomide resistance [56,189]. Disruption of *DDB1*, which is another subunit of the CRL4^CRBN^ ubiquitin ligase has also been associated with lenalidomide resistance [56]. Additionally, loss-of-function of the *CSN9* signalosome complex might activate the SCFF^bxo7^ complex, enhancing the degradation of CRBN and, consequently, conferring resistance to lenalidomide and pomalidomide [56,189]. Therefore, the downregulation of *CRBN* expression and attenuation of neosubstrate degradation has appeared to be the major mechanism of IMiD resistance [69]. In order to identify differences between the two IMiDs, a later study has focused on the non-overlapping hits, highlighting that the inactivation of *NCOR1*, *EDC4*, *SCAP*, *UBE2G1*, or *MBTPS1*/2 could confer selective resistance to lenalidomide but not to pomalidomide [69]. Another study has determined that the top hits from CRISPR KO studies conferring resistance to PROTACs (proteolysis-targeting chimeras) operating via CRBN were CRBN itself and, to a quantitatively lesser extent, other members or regulators of the Cullin 4A–RING–CRBN ligase complex (CRL4CRBN) that catalyzes the ubiquitination of target(s) for degronimids. Similarly, components or regulators of the CRL2VHL complex, including CUL2 and VHL themselves were the top hits operating via VHL [190].

Taking into account that current treatment regimens for newly diagnosed MM consist of a combination of an IMiD, PIs, glucocorticoids and chemotherapy [191], multidrug therapy makes it challenging to infer the impact of distinct gene mutations on the activity of each drug. The integration of exome data from pretreatment and relapsed MM samples and functional CRISPR-based screens have determined the role of genes on the efficacy of the four most frequently used drugs (bortezomib, lenalidomide, dexamethasone, and melphalan) in MM treatment. It is worthy to mention that CRISPR screens for each drug have revealed genes whose inactivation caused drug-specific resistance, with little overlap between the tested drugs, suggesting non-relevant cross-resistance among these drugs [91]. These included CRBNE3 ligase complex members (*CRBN*, *DDB1* and *CUL4*) for lenalidomide—as other studies have shown [56,69,189]—structural genes (*PCDHA5* and *ANKMY2*) or NF-κB pathway-related genes (*BIRC3* and *TRAF3*) for dexamethasone, cell cycle regulators (*RB1* and *CDK2NC*) for BTZ, and DNA damage repair genes (*TP53*) for melphalan [91].

### 4.2. Myeloid Neoplasms

#### Acute Myeloid Leukemia

AML is a malignant disease characterized by the clonal expansion and differentiation arrest of bone marrow myeloid progenitor cells [192]. Chemotherapeutic agents such as cytarabine (Ara-C) are the backbone of the standard of care for AML [193]. Despite their efficacy, a major proportion of AML patients has shown chemotherapy resistance and relapse, being the most difficult challenges to cure [194]. Many different studies have tried to elucidate Ara-C resistance mechanisms to find more effective strategies [195]. Particularly, genome-wide CRISPR-knockout screens not only have confirmed the role of *DCK* in Ara-C resistance (previously reported to be downregulated in resistant AML cell lines [196]), but also have uncovered a novel secondary candidate *SLC29A* [49]. Of note, both genes have been implicated in cytarabine uptake and the consequent metabolic activation of Ara-C in treated cells. Alternatively, an independent CRISPRa approach has also confirmed the implication of *DCK* and other relevant genes for cytarabine metabolism (*ENT1* and *CDA*) [40]. As a further step, the CaLR strategy has elucidated for the first time the significant implication of some lncRNAs, such as *GAS6-AS2*, during chemotherapy resistance [40]. 

As one-third of newly diagnosed AML patients are characterized by the presence of *FLT3* mutations, which is also overexpressed on the majority of AML blasts [197], FLT3 inhibitors (FLT3i) such as midostaurin, gilteritinib and sorafenib have been implemented into clinical practice. Although FLT3i have significantly improved survival rates of *FLT3*-mutated AML patients, FLT3i resistance has become an unmet medical need [198]. Genome-wide CRISPR screening has been applied for the study of sorafenib profile responsiveness, identifying the loss of the negative regulators (*LZTR1*, *NF1* and *TSC2*) of MAPK and MTOR pathways during drug resistance [70]. A similar approach has determined the role of *SPRY3* (a known inhibitor MAPK signaling) and *GSK3A* (canonical Wnt signaling antagonist) as being the main mechanisms of FLT3i resistance [48]. Consequently, the combination of MEK inhibitors and FLT3i have shown enhanced efficacy [199]. In addition, *BCL2* overexpression has been widely described in relapse/refractory (R/R) AML patients; therefore, the BCL2 inhibitor venetoclax has received the FDA approval for AML treatment in 2018 [200]. To study the mechanisms underlying venetoclax resistance, two independent screening using two known genome-wide sgRNA libraries have determined, with high degree of confidence, the implication of mitochondrial regulators and effectors of apoptosis in this drug resistance, highlighting *TP53*, *BAX* and *PMAIP1* as their top hits [72]. As aforementioned, similar pathways during venetoclax resistance have been detected in CLL [63]. Along the same lines, other genes involved in mitochondrial organization and function have been described in venetoclax resistance, such as the mitochondrial chaperonin *CLPB*, or the cristae shape *OPA1*. Then, targeting mitochondrial architecture may provide novel strategies to overcome venetoclax resistance in AML [61].

Since AML remains an incurable disease for the majority of patients, great efforts have focused to develop novel inhibitors during the last decade [201]. In this sense, CRISPR screening studies have helped to anticipate potential mechanisms of resistance for some of these new therapeutic approaches in AML [44,73,120,202].

## 5. Hematologic Dependency Map through CRISPR Screens: Essential Genes and Key Regulators of Drug Sensitivity

Nowadays, there is a huge effort in mapping the landscape of cancer vulnerabilities in all tumors, including the hematological neoplasms, by the application of high-throughput CRISPR–Cas9 genetic screens [131,149,203]. These technologies allow us to identify essential genes for cellular survival. Those ones which become specific gene dependencies in a few cell types of hematological neoplasms might represent better drug targets than common cancer essential genes since inhibiting their function is less likely to cause toxicity. As a further step to improve personalized medicine, genes whose function is selectively essential in the context of a particular genetic aberration represent promising targets for the development of precision therapeutics by synthetic lethal effects. In addition, in the presence of drug exposure, CRISPR negative screens are able to identify genetic biomarkers of drug sensitivity and genes that could be modulated to increase the efficacy of one drug [204].

### 5.1. Lymphoid Neoplasms

#### 5.1.1. Lymphoma

Several studies have revealed lymphoma-specific gene dependencies using CRISPR high-throughput analysis. The application of a CRISPR knockout library in different DLBCL cell lines has broadly uncovered potential genes essential to the development and maintenance of lymphomas [52]. Thereby, 1956 “DLBCL essential genes” whose inhibition resulted in significantly decreased cell fitness were described, including the oncogenes *MYC*, *RHOA*, *SF3B1*, *MTOR*, and *BCL2.* By contrast, *TP53*, *MGA*, *PTEN*, and *NCOR1* were positively enriched in the screen, playing a role as tumor-suppressor genes. Interestingly, nine out of all of the oncogenes, which have been reported as direct targets of drugs, are in clinical trials or already in use for another indication. In this study, gene dependencies of each subtype-specific DLBCL entity were also described: the knockout of *EBF1*, *IRF4*, *CARD11*, *MYD88*, and *IKBKB* were selectively lethal in activated B-cell (ABC) DLBCL, whereas *ZBTB7A*, *XPO1*, *TGFBR2*, and *PTPN6* were lethal in germinal-center B-cell-like (GCB) DLBCL. Employing a similar approach in ¡BL cell line, the inhibition of hydroxymethyltransferase 2 (*SHMT2*), a key enzyme in one-carbon metabolism, was shown as a potential therapeutic vulnerability [205]. Likewise, genes encoding AP-1-family transcription factors (*BATF3* and *JUNB*), regulators of the G1-S phase cell cycle phase transition (*CDK6* and *CCND2*), components of the JAK/STAT pathway (*STAT3* and *IL10RB*), RNA-binding proteins (*SYNCRIP* and *ZFP36L2*), and a protein prenyltransferase subunit (*PTAR1*) were described as essential in adult T-cell leukemia/lymphoma (ATLL) [65]. Taking into account these results, the CDK6 inhibitor palbociclib may be an effective strategy to treat ATLL [65].

In recent years, several specific genetic alterations with clinical implication have been described in lymphomas [206,207,208]. Therefore, the search for synthetic lethality in these different genetic backgrounds could represent great treatment advances for these specific groups of patients. That is the case of the histone acetyltransferases (HATs) *CREBBP* and *EP300* mutations, which contribute to a worse prognosis of DLBCL and disease relapse [209]. Nie et al. have revealed a synthetic lethal interaction between *CREBBP* and *EP300*, concluding that HAT inhibition could be a promising therapeutic option for *CREBBP-* or *EP300*-mutated DLBCL [122]. Another key genomic alteration related with high-grade B-cell lymphoma prognoses are *MYC* translocations [210], which occur in 80% of BL [211] and 10% of DLBCL [212]. An in vivo CRISPR approach has been performed in a transgenic murine model with *Myc* rearrangements, Eμ-myc, to identify and validate rare tumor suppressors such as *Sp3* or *Phip* that could accelerate lymphomagenesis in the context of *Myc* translocation [110]. Finally, TNFAIP3 (A20) is a ubiquitin-editing protein whose inactivation by nonsense/deletions or missense mutations is a recurrent genetic alteration of HL cases [213]. A comprehensive understanding of how the ubiquitination pathway regulates HL pathogenesis in deficient-A20 HL has identified the essential role of TAK1 kinase by the use of a custom unique ubiquitin regulator-focused CRISPR library. Interestingly, the TAK1 inhibitor takinib has shown promising activity against HL in vitro and in vivo models [89].

Moreover, the search of genes and pathways that mediate sensitivity to drugs has been widely explored using CRISPR libraries in lymphoma diseases. Of note, *BLNK* and *BTK* genes, key components of the BCR signaling, have been described as pivotal modulators of the sensitivity of rituximab that is contained in the standard care R-CHOP chemotherapy regimen for DLBCL [53]. Precisely, inhibitors of the BCR pathway such as ibrutinib have been approved in B-cell lymphomas [214,215]. Taking into account that only a subset of DLBCL patients benefited from ibrutinib, both genome-wide and targeted follow-up screens have discovered a multiprotein supercomplex, formed by *MYD88*, *TLR9* and the *BCR*, that could be associated with ibrutinib responsiveness [68]. Similarly, the BCL2 network was determined as a key mediator of the sensitivity of luxeptinib (dual BTK/SYK inhibitor), suggesting a synergy of luxeptinib and venetoclax [76]. Furthermore, sensitivity modulators of IMiDs drugs, which represent an emerging treatment of PEL lymphoma, have been also elucidated, highlighting *MYC*, *IRF4* and *CK1α* [66]. In another study, *IKZF1* was identified as a top candidate for sensitizing DLBCL cells to tazemetostat, an *EZH2* inhibitor. This drug has a synergistic effect with lenalidomide through epigenetic modulation [216]. Lastly but not least, huge efforts are coming together to decipher the sensitive genetic regulators of novel targeted drugs [54,88,97,216,217] as well as antibiotics [51] in different B and T-cell non-Hodgkin and Hodgkin lymphomas with the aim of exploring novel combinatorial therapeutic approaches.

#### 5.1.2. Acute Lymphoblastic Leukemia

Few studies have been focused on the evaluation of genetic dependencies of B-ALL. In fact, although B-ALL is the most common type of leukemia in the pediatric population, the first-generation pediatric cancer dependency map only includes solid tumors [218], and therefore, further genetic screenings should identify pediatric liquid tumor dependencies to complete the map. On the other side, two CRISPR-based genetic screens (CRISPRa and CRISPRko) have been performed to measure the mechanistic contribution of *PAX5* target genes to leukemic cell growth, since the abnormal expression of this transcription factor has been widely observed in B-ALL patients. Their results have shown that the deletion of the *PAX5* targets, such as *NR3C1*, *TXNIP* and *CNR2*, provided a strong survival advantage verifying their tumor-suppressor function through the transcriptional repression of glucose transport and the restriction of metabolites [219]. Regarding the T-ALL subtype, preTCR pathway genes have also been identified as genetic dependencies thanks to the application of genome-wide CRISPR screens [77].

Specially, highly aggressive forms of B-ALL such as those harboring *MLL-AF4* translocations need to be better characterized in order to outline potential therapeutic targets with a high specificity. Interestingly, CRISPR screening was performed to determine RNA-binding proteins as being specific essential genes for MLL-AF4-dependent leukemic cell growth, validating *USO1* as a vulnerability in in vitro ALL models and primary murine cells [80].

Intensive chemotherapy combined with other drugs can be curative in ALL. Taking advantage of CRISPR genome-wide techniques, multiple genes whose inactivation increases drug activity have been discovered across seven ALL chemotherapy drugs. Among these, *PPM1D* and *BCL2* have been scored broadly as common targets whose inhibition could enhance the response to vincristine, 6-MP, L-asparaginase, Ara-C, daunorubicin and maphosphamide. A PPM1D inhibitor and BCL2 inhibitor have enhanced the antileukemic effect of chemotherapy in relapsed ALL xenograft samples ex vivo [42]. Another CRISPR-based screen of kinases has only focused on one of these chemotherapy drugs, ASNase therapy, to identify its sensitivity regulators. Among them, *BTK* was a significant gene-sensitization factor that could be pharmacologically inhibited by ibrutinib to strongly synergize with ASNase to induce leukemic cell death [27].

#### 5.1.3. Chronic Lymphocytic Leukemia

As CLL is a clinically and genetically heterogeneous disease [220,221], several studies have analyzed, using CRISPR technology, the cellular impact of the most recurrent genetic aberrations [222,223,224,225]. However, there are no studies with the aim of characterizing the CLL-specific essential genes by genome-wide CRISPR screenings in an unbiased way. Only one custom CRISPR screen approach of CLL-related transcription factors (TFs) has uncovered *PAX5*, *IRF4*, *EBF1* and *BATF* as the most significant essential TFs [86]. Interestingly, BET inhibitors such as JQ1 were proposed as an effective strategy to target CLL cell growth, since they downregulated the expression of essential TFs (*PAX5*, *MYC, IKZF1* and *RARA)* [86].

Another potential therapeutic alternative for CLL are PARPi [226], which are, specifically, more effective in CLL cells harboring *ATM* alterations [224,227]. As an additional step, CRISPR screening has identified synthetic lethality in deficient-*RNASEH2A* or *RNASEH2B* cells. Since *RNASEH2B* is located in a 13q deletion, which is the most common chromosomal alteration in CLL, PARPi could be beneficial for patients carrying these alterations [79].

#### 5.1.4. Multiple Myeloma

Genomic studies have revealed which genes may play important roles in the pathogenesis and progression of MM [228,229]. As a further step, CRISPR–Cas9 knockout in vitro screens have been useful to identify potential MM-specific essential genes such as *TRAF3* and *FAM46c* [91]. Since the bone marrow microenvironment is essential for MM disease establishment and progression [230], targeted CRISPR screening was applied in an in vivo model discovering *HMGA1* and *PA2G4* as key determinants in myeloma progression and as potential therapeutic targets [92].

The functional association of specific genetic alterations with drug sensitivity will help to personalize the treatment of MM in the future. In this sense, the CRISPR high-throughput approach has allowed us to uncover that the inactivation of genes involved in the DNA damage repair pathway, including *ATM*, *FANCA*, *RAD54B*, and *BRCC3*, enhances susceptibility to cytotoxic chemotherapy [91]. Regarding IMiD sensitivity, proteasome inhibitors or NAE inhibitors could suppress CRBN degradation and, thereby enhance sensitivity to IMiDs [189] and the loss of TOP2B resensitized IMiD-refractory MM cells [69].

### 5.2. Myeloid Neoplasms

#### 5.2.1. Myelodysplastic Syndromes

Myelodysplastic syndrome (MDS) is a myeloid lineage malignancy characterized by ineffective hematopoiesis and risk of evolution to AML [231]. The heterogeneous nature of MDS demands a personalized variety of therapeutic approaches [232]. In the search of novel therapies, genome-wide CRISPR knockout screening has identified the U2 spliceosome complex as an essential factor to promote nonsense-mediated RNA decay (NMD) [55]. Interestingly, these findings have suggested NMD as a particular therapeutic vulnerability in MDS cells with mutations in the U2 spliceosome genes *SF3B1* and *U2AF1* which have been recurrently detected in MDS patients [233].

Moreover, there are promising treatments such as rigosertib in clinical trials [234]. To unsettle its mechanism of action, Jost et al. performed a chemical-genetic strategy that combines CRISPRi and CRISPRa screens, revealing that rigosertib sensitivity is affected by microtubule-associated genes such as *TACC3* and *KIF2C* [36].

#### 5.2.2. Acute Myeloid Leukemia

The first work that has ever used genome-wide CRISPR libraries to characterize essential genes in a hematological malignancy was performed in a series of AML cell lines. This work has identified a total of 492 specific essential genes for cell survival in five different AML cellular models, with 33 clinically actionable candidates such as *BCL2*, *DOT1L* or *BRD4* [32]. Specifically, among the differentially essential genes, *KAT2A* was highlighted as a potential druggable gene since its disruption only affected AML cell survival, but not normal progenitors. Another study was focused on the role of *TAZ* (mitochondrial transacylase) as an AML-gene dependency [119]. TAZ was also observed as an essential gene for the growth in all the AML cells tested in the previous aforementioned work [32]. Unlike to these studies performed with human cell lines, others have tracked genetic vulnerabilities in mouse AML cell lines, identifying not only well-defined genes involved in leukemogenesis (*Kras*, *Nras*, *Bcl2l1*, *Jak1*, *Jak2*, *Brd4* and *Brd9*), but also potentially actionable targets such as *Dcps.* Since this gene interacts with components of the spliceosomes, its inhibition could be particularly sensitive in AML patients harboring spliceosome mutations [25]. Using an alternative microRNA-knockout CRISPR library, 10 AML-essential miRNAs were uncovered, highlighting *miR-19b-1* and *miR-19b-2* because they are commonly involved in signal transduction, apoptosis, TGF-beta and MAPK pathways [81]. More recently, a surface antigen-based CRISPR platform has discovered new regulators of leukemia differentiation such as *ZFP36L2* whose disruption promotes AML differentiation [235]. In vivo CRISPR screening platforms have also successfully revealed several targets in AML, such as *SLC5A3*, which is implicated in metabolism, or *MARCH5,* which is related to apoptosis pathway [82]. 

Other authors have used publicly available data to identify essential factors for AML growth. In this sense, *IRF8* was uncovered as an AML-specific susceptibility factor [83]. Using data uploaded in DepMap (https://depmap.org/portal/, accessed on 25 June 2022), the glutamine–cysteine ligase catalytic subunit (*GCLC*) was extracted as a fitness gene in AML cell growth, survival, clonogenicity and leukaemogenesis [84]. Similar datasets in combination with druggability data published a list of 44 out of 94 AML-essential genes that could be targeted by existing or experimental drugs [78].

In the context of *MLL* rearrangements which characterize an aggressive subtype of the disease [236], *HATs* of the MYST family were shown as fitness genes [237]. In particular, the lysine acetyltransferase *KAT7*, which is derived from the *MLL-X* fusion gene, has been identified as a genetic vulnerability of this AML subgroup subtype [237]. Notably, in an AML cell line harboring *MLL* rearrangements, *ENL* was described as a particular essential dependency for proliferation. Other researchers identified *POLR2M* or *PAICS* as potential therapeutic targets for this type of AML [46,47]. Furthermore, in *FLT3*-ITD-positive AMLs, CRISPR screening has revealed *Kdm1a*, *Brd3*, *Ezh2* and *Hmgcr* genes as promising targets in this genetic context [74]. In another AML entity characterized by the presence of *RAS* mutations which have been associated with dismal prognosis [238], synthetic lethal genes involved in the maturation of the Ras and MAPK signaling pathway were required only in the context of oncogenic Ras [239]. Finally, some interest has also been shown in tracking the vulnerabilities in *CUX1*-deficient AML models, describing *CFLAR* as a selective therapeutic candidate [75].

The combination of potent drugs has achieved higher rates of complete response and long-lasting remission in AML [240]. Some efforts have focused on improving the efficacy of FLT3i by targeting signaling pathways. In this regard, genome-wide loss-of-function screening has highlighted that *XPO1* knockdown cells were more sensitive to the FLT3is midostaurin and gilteritinib. Consequently, the combination of selinexor (XPO1 inhibitor) plus midostaurin or gilteritinib could significantly improve survival in an AML xenograft mouse model [62]. Similar CRISPR screening has uncovered that BCL2 inhibition has also enhanced FLT3i antitumoral activity [241] and its combination with FLT3i was proposed for treating AML. In a similar way, the first enzyme in glutamine metabolism, GLS, was described as being synthetically lethal with FLT3i [71]. Regarding BCL2 inhibitor response, synthetic lethal partners were also described to improve venetoclax sensitivity, pointing out those involved in mitochondrial cristae maintenance and function [61]. Similar approaches have also revealed metabolic genes capable of influencing cellular commitment to apoptosis and sensitizing AML cells to venetoclax [82,85]. Finally, CRISPR high-throughput approaches have anticipated gene determinants to enhance the sensitivity of small molecules under development for AML treatment [45,73,242].

#### 5.2.3. Chronic Myeloid Leukemia

The clinical management of chronic myeloid leukemia (CML) has been impressively improved by tyrosine kinase inhibitors that target Abl kinases [243]. However, these inhibitors are not effective when the disease is in the accelerated or blast crisis phase [244]. In this context, genome-wide CRISPR-based studies have helped to comprehensively map the biological regulators of blast crisis CML. An in vivo CRISPRko screening has identified RBPs as being regulators of aggressive myeloid leukemia progression [109]. Among them, *Stau2* was further validated as a critical novel regulator on blast crisis CML initiation and propagation. 

Although tyrosine kinase inhibitors such as imatinib have been effective to treat CML patients, resistance to this targeted therapy still represents a complex multifactorial process and a challenge for clinicians [245]. An alternative approach based on the CRISPR-based double knockout (CDKO) system was applied in the CML K-562 cell line to discover novel synergistic drug combinations, highlighting that the dual inhibition of *BCL2L1* and *MCL1* may be an effective way to combat resistance in CML [87]. Interestingly, another study was performed using a custom library containing sgRNAs disrupting MYC binding sites in order to identify MYC-dependent vulnerabilities involved in the development of CML [64]. Due to tyrosine kinase inhibitor resistance, alternative therapeutic approaches such as arsenic trioxide and interferon alpha have been explored in human primary CML cells [246]. Genome-wide CRISPR/Cas9 screening has reported gene determinants whose loss may confer either increased sensitivity to arsenic trioxide [50].

## 6. Other Applications of CRISPR Screenings

CRISPR screens can be applied to reach other goals and overcome other current challenges in cancer [11]. Interestingly, pooled CRISPR libraries have been used to report metastasis-related genes in solid tumors [108,247]. In the field of hematological malignancies, CRISPR studies have been focused on deciphering the molecular mechanisms of the transformation syndromes [134,248]. It is worthy to mention that disease transformation still represents a challenge for clinical management in patients with clonal hematological disorders [249]. In particular, 30% of patients with MDS develop secondary AML during the course of the disease and the mechanisms of disease progression from a chronic MDS phase to a more aggressive AML phase are still poorly understood [250]. Genome-wide loss-of-function CRISPR/Cas9 screens have reported the loss of *FBXO11* as a mechanism for myeloid transformation since its inhibition confers cytokine independent growth to MDS cells [134]. Of note, mutations in this gene have been detected in de novo AML patients suggesting a tumor suppressor role for *FBXO11* in myeloid malignancies [251]. Other disorders that can potentially transform to AML aggressive disease are MPNs. Although several NGS studies have identified gene mutations in post-MPN AML patients [252,253], the mechanisms of this transformation remain largely unclear [254]. Recently, the combination of two custom loss-of-function mouse libraries have revealed Lkb1 and Stk11 kinases as being novel drivers of this leukemic transformation [248]. Therefore, the aforementioned CRISPR screenings have provided insights into potential gene targets to improve the clinical management of secondary AML patients [134,248].

Another powerful application of high-throughput CRISPR technology is the assessment of virus-associated oncogenesis mechanisms [255]. Particularly, since Epstein-Barr virus (EBV) can cause endemic BL and other immunosuppression-related lymphomas [256], CRISPR genome-wide screens have been parallelly performed in BL and lymphoblastoid cell lines to identify several EBV-driven B-cell synthetic lethal targets for therapeutic intervention, including the evasion of TNFα, BIM, and BLIMP1 effects [257]. In this context, another CRISPR study has gained insight into the epigenetic mechanisms with the aim of restricting the expression of EBV immunogenic oncoproteins [258], finding *UHRF1* and its DNA methyltransferase partner *DNMT1* as being critical factors for viral transformation. Similar approach has been also applied in PEL to decipher the transformative properties of Kaposi’s sarcoma-associated herpesvirus [259]. This approach has discovered that PEL cell lines are strongly dependent on *CCND2* and *MCL1* expression which makes them highly sensitive to palbociclib or S63845 drugs.

Furthermore, there is a huge interest in the promising applications of CRISPR-editing technology in the field of cancer immunotherapy [260,261]. In recent years, the ongoing development of new and effective immunotherapies presents important therapeutic opportunities in hematological malignancies [262]. Immune checkpoint inhibitors have led to important clinical advances [263]; however, the underlying resistance and immune escape mechanisms are not wholly understood [264]. A CRISPR screen study performed in anaplastic large-cell lymphoma with exposure to the anti-PD1 antibody nivolumab has pointed out the role of *STAT3* and as well as *GRB2/SOS1*, which activates the MEK/ERK and PI3K/AKT signaling pathways, as novel new targets to prevent PD-L1-mediated tumor immune escape [265]. Moreover, taking into account that bispecific antibodies represent a novel approach for NHL treatment [266], an activation screening has concluded that surface proteins involved in cell–cell adhesion (SPN, CD25 and MUC1) could limit CD20xCD3 antibody-mediated tumor cell killing [267]. Furthermore chimeric antigen receptor-modified (CAR) T cells targeting CD19 have revolutionized the treatment of B-cell malignancies increasing cure rates [268]. A better understanding of which mechanisms can influence CAR T-cell cytotoxicity and resistance could improve current cellular immunotherapies. Interestingly, CRISPR-library screening has garnered a better understanding of the mechanisms of anti-CD19 CAR-T cell cytotoxicity, mainly in ALL and lymphoma, uncovering potential pathways, such as death receptor signaling as a key mediator of CAR-T efficacy [124,269,270]. More recently, the loss of *NOXA*, a BCL2 family member, has been also reported as a pivotal regulator of resistance to CAR T-cell therapy in B-cell malignancies thanks to the application of CRISPR screening approaches [271]. Moreover, CAR-BCMA T cells have achieved substantial activity against heavily treated relapsed/refractory MM [272] but resistance to BCMA-targeted CAR-T therapies has happened in some MM patients through the downregulation or loss of the targeted antigen [273]. The combination of CRISPRi/CRISPRa functional genomics approaches have identified both antigen-dependent (*HDAC7, SEC61A1*) and antigen-independent pathways (*GALE*, *GNE*) that could control the response of MM cells to BCMA-targeted CAR-T-cells [123]. In terms of myeloid neoplasms, CRISPR screening has uncovered the genes that sensitize AML cells to double-negative T-cell therapy, highlighting *CD64* as a predictive marker for response to this treatment in AML patients [274].

## 7. Conclusions and Future Perspectives

**Figure 2:**
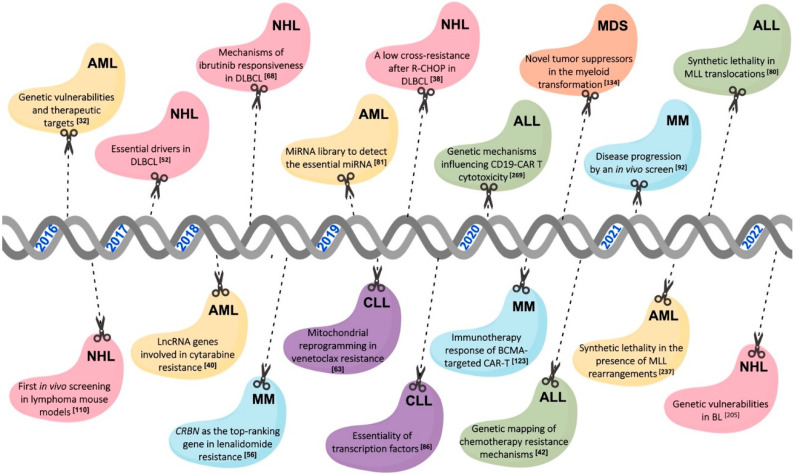
Most relevant CRISPR-screening studies in the field of hematology, discussed in this review. The double strand of DNA represents a temporary line. The different hits are represented inside a Cas nuclease scheme in chronological order and classified by diseases: AML, acute myeloid leukemia; NHL, non-Hodgkin-Lymphoma; CLL, Chronic lymphoblastic leukemia; MM, multiple myeloma; ALL, acute lymphoblastic leukemia; MDS, myelodysplastic syndrome.

CRISPR screening provides a high-throughput way for functional genomic studies in hematological malignancies. It has been applied in the study of different blood cancers with the aims of the identification of novel modulator genes of drug efficacy, therapeutic targets, synthetic lethal interactions, biomarkers of malignant transformation and genetic determinants of immune evasion. Some of the most relevant CRISPR-screening studies in the field of hematology, which have been discussed in this review, have been highlighted in Figure 2. This technology not only is anticipated to determine the resistance mechanisms to novel drug such as immunotherapies, but also has been deeply explored for the discovery of drug targets as potential treatments in hematological diseases such as in AML or NHL. Despite the huge amount of the data obtained from the application of CRISPR libraries, only a few candidates have been validated for each study. Further experiments might give deep insight into other significant hits which do not achieved the top score, but they could play a key role and even could be commonly detected in different screenings with a similar purpose. Therefore, great efforts should focus on the integration and harmonization of the data obtained from the application of CRISPR screening approaches [150]. 

We have reviewed the results related to the drug resistance mechanisms discovered in hematological neoplasms thanks to CRISPR screening. Since some drugs are commonly used across different blood diseases, it is worthy to mention that similar mechanisms of drug resistance have been described independently to the disease (Figure 1). Interestingly, the loss-of-function of the *BAX* gene has been commonly involved in venetoclax resistance in both CLL or AML diseases [63,72], *DCK* disruption in the resistance of cytarabine for AML or ALL [42,49] and *CSN* subunits of the CSN9 complex have been related with IMiDs resistance in MM or lymphoma [56,90,189]. Moreover, we can anticipate the resistance mechanisms of drugs under investigation through CRISPR approaches before their approval and the molecular characterization of a cohort of patients treated with novel drugs (for example, BET inhibitors, HSP90 inhibitors…) [44,73,120,202]. Taking into account that novel therapies are continuously emerging in hemato-oncology, we therefore envision that CRISPR screening will continue to have a role in the era of targeted therapy and immunotherapy. Lastly, the assessment of combinatorial loss-of-function or activation screenings and the implementation of in vivo CRISPR screening and single-cell CRISPR perturbations in blood cancer models will allow us to improve the characterization of drug mechanisms of action in order to delineate genes and pathways that govern drug response.

It has been proposed a list of essential genes in a few diseases such as AML, DLBCL and MM using different cellular or in vivo models [32,52,92]. Among the fitness genes, some of them be druggable targets (*KAT2A* in AML). More examination is needed in this way in order to generate a comprehensive hematologic dependency map which will suggest novel therapeutic vulnerabilities for the non-studied up-to-date diseases. In addition, since large-scale sequencing studies have determined the genetic alterations with clinical implication in different hematological diseases, synthetic lethalities have deciphered the essential genetic interactions in aggressive disease entities of AML or DLBCL through CRISPR approaches [80,122]. Therefore, this technology will open the door to search novel synthetic lethalities for ongoing precision medicine clinical trials in other well-known genetic neoplasms such as MM or CLL. 

## Figures and Tables

**Table 2:** Bioinformatic tools for CRISPR screening in hematology.

Name Algorithm	Brief Description	Original Purpose	Software	CN Correction	Guide Inefficiencies	Visualization Tools	Type of Library in Hematology	Applications in Hematological Neoplasms
**MAGeCK** [114,116,117,118]	Binomial model method that prioritizes sgRNA, genes and pathways. MAGeCK–VISPR and MAGeCK–Flute are updated versions that provide advantages such as QC analysis, visualization or CN correction while scMAGeCK is a version specifically adapted for single-cell sequencing data.	CRISPRko	Python, R	Yes	Yes	Yes	CRISPRko, CRISPRa, CRISPRi	**AML** [25,32,47,62,70,71,72,74,81,119,120,121], **NHL** [39,52,53,65,67,76,90,97,122], **HL** [51], **CLL** [79], **MM** [91,123], **ALL** [27,41,42,43,124], **MDS** [55]
**STARS** [28]	A method based on a gene-ranking system that calculates gene scores using a binomial model.	All CRISPR screens	Python	No	No	No	CRISPRko	**ALL** [125], **LLC** [63], **MM** [56]
**BAGEL** [126,127]	Supervised learning method for analyzing CRISPR knockout screens which uses the fold changes of all gRNAs targeting all genes and core essential and nonessential gene lists to estimate an essentiality factor.	CRISPRko	Python	No	No	No	CRISPRko	**AML** [75,128], **CML** [129]
**casTLE** [130]	Maximum Likelihood Estimator that combines measurements from multiple targeting reagents to estimate a maximum essentiality effect size and a *p*-value.	CRISPRko, CRISPRi, CRISPRa and RNAi	Python	No	No	Yes	CRISPRko	**CML** [87]
**CERES** [131]	A method that estimates gene dependency levels in multiple CRISPR essentiality screens while correcting the CN specific effect.	CRISPRko in multiple screens	R	Yes	Yes	No	CRISPRko	**AML** [128]
**JACKS** [132]	Bayesian method that models gRNA efficacies in multiple screens performed with the same sgRNA library.	CRISPRko in multiple screens	Python	No	Yes	No	CRISPRko	**AML** [128]
**PinAPL-Py** [133]	A method that develops a full automated workflow for CRISPR screening analysis.	All CRISPR screens	Web-based (http://pinapl-py.ucsd.edu, accessed on 25 June 2022)	No	No	Yes	CRISPRko	**MDS** [134]
